# Long-Term Surgical Outcomes of Carotid Body Tumors With Pathological Fibrosis: A Cohort Study

**DOI:** 10.3389/fonc.2021.684600

**Published:** 2021-07-19

**Authors:** Hanfei Tang, Xiaolang Jiang, Song Xue, Weiguo Fu, Xiao Tang, Daqiao Guo

**Affiliations:** Department of Vascular Surgery, Institute of Vascular Surgery, Zhongshan Hospital, Fudan University, Shanghai, China

**Keywords:** carotid body tumor, fibrosis, cranial nerve injuries, tumor recurrence, progression-free survival

## Abstract

**Objective:**

To compare the surgical outcomes of carotid body tumor (CBT) with or without pathological fibrosis, and evaluate the associated factors of fibrous CBT (FCBT).

**Materials and Methods:**

Paraffin-embedded tissues of 236 patients with unilateral CBTs at our center were retrospectively reviewed from January 2008 to May 2020. Based on the pathologic features, CBTs were divided into FCBT and conventional CBT (CCBT) groups. The clinical data and surgical outcomes of the two groups were compared.

**Results:**

Of 236 patients, 53 had FCBT and 183 had CCBT. FCBTs showed higher vascular invasion (24.53%), marked pleomorphism (22.64%), internal carotid artery reconstruction (37.74%), estimated blood loss (559.62 cm^3^), and postoperative nerve injury (49.06%), with lower 10-year recurrence- (89.2%) and major adverse event-free survival (87.3%) compared to CCBTs. Nerve injury was correlated with the Shamblin grade; major adverse events and nerve injury were both correlated with pathological fibrosis.

**Conclusion:**

Compared with CCBT, FCBT is prone to increased recurrence, metastasis, major adverse events, and nerve injury risk. Early surgical resection, routine excision of surrounding abnormal lymph nodes, and closer clinical surveillance in FCBT patients are recommended.

## Introduction

Carotid body tumors (CBTs) are typically benign, slow-growing tumors arising from neuroectodermal crest-derived paraganglia (1–3). CBTs appear clinically as a slowly enlarging, painless mass displaying a characteristic histological “Zellballen” pattern composed of nests of uniform cells separated by numerous blood capillaries (4).

CBTs can be classified by etiology, anatomy, and genetic mutations. The three recognized etiological types are sporadic (the most common), familial, and hyperplastic. Hyperplastic CBTs are commonly diagnosed in the context of chronic hypoxia, for example, in patients living at high altitude or those with chronic lung disease. The anatomical classification by Shamblin et al. (3), designed as a predictor of intra-operative technical difficulty (5), is the most clinically relevant, because it describes the extent of envelopment of the common carotid artery (CCA), internal carotid artery (ICA), and external carotid artery (ECA) by CBT. Genetic carriers of *subunits of succinate dehydrogenase* (*SDHx*) mutations present a higher risk of tumors for the autonomic nervous system. However, tumor function, age of onset, risk of malignant diseases, and transmission vary by genotype (6, 7).

CBTs have occasionally exhibited unusual morphology, including clear cells, spindling of cells, and angiomatoid features (8). In 2006, Plaza et al. (8) reported a series of patients with paraganglioma with extensive fibrosis, an uncommon pattern of growth that closely resembled an invasive malignant neoplasm (9). However, to the best of our knowledge, the clinical significance of pathological fibrosis in CBT has never been investigated.

Therefore, we compared the procedural details, tumor characteristics, postoperative complications, and outcomes in fibrosis CBT (FCBTs) and conventional CBT (CCBT), to ascertain the role of pathological fibrosis in surgical outcomes of CBT.

## Methods

### Patient Selection

Paraffin-embedded tissue of CBT from 261 individual patients were obtained from surgical pathology archives of our center between January 2008 and May 2020. Of these, 23 patients with bilateral CBT and two patients with recurrent tumor before resection were excluded. Patients’ demographics, radiologic findings, and operative interventions were reviewed. This study was conducted in accordance with the Declaration of Helsinki. An approval was obtained from the Institutional Review Board. All research participants provided written informed consent.

### Pathologic Features

**Figure 1:**
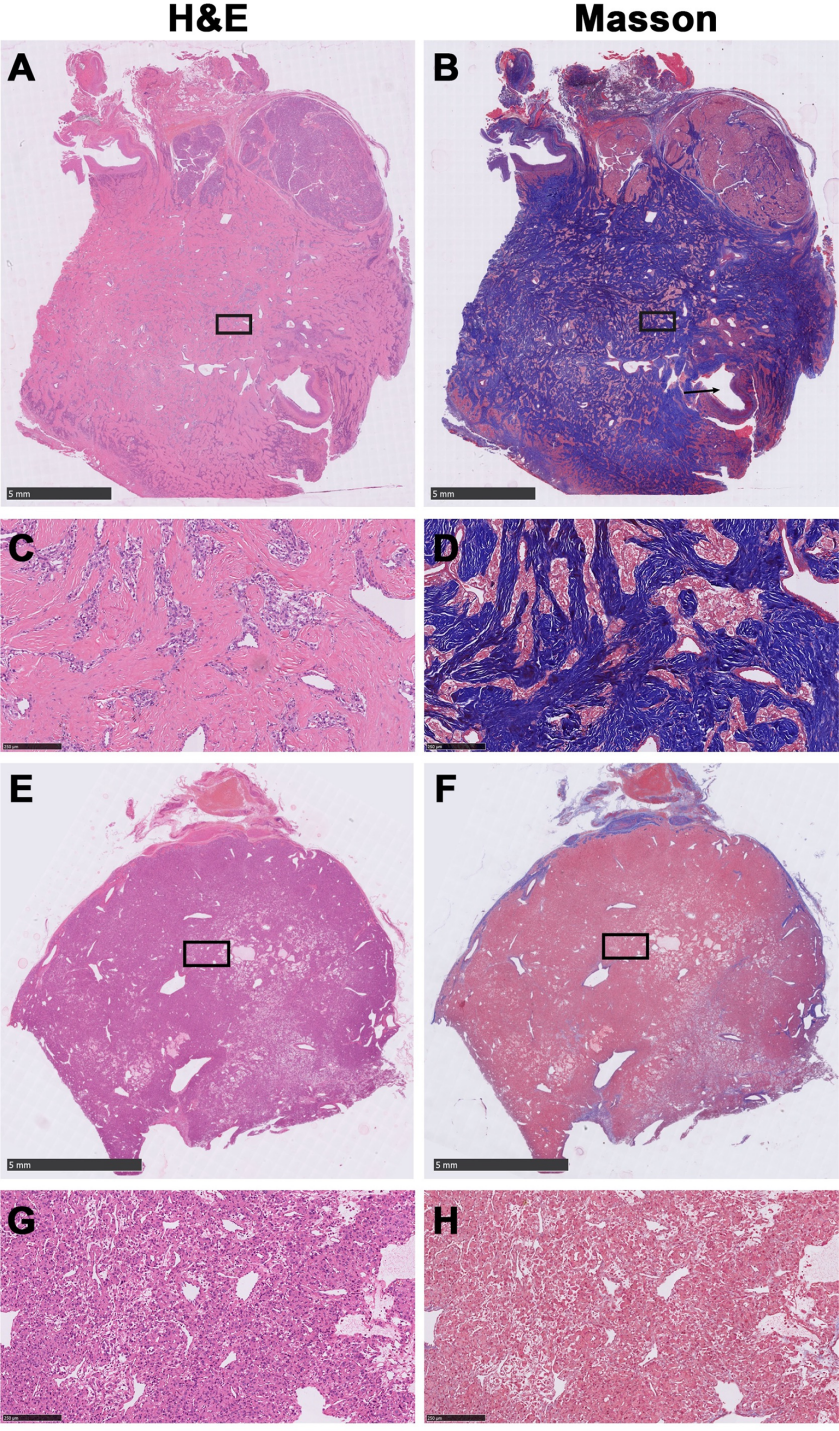
Representative histological images of fibrous carotid body tumors (FCBTs) and conventional carotid body tumors (CCBTs). FCBTs showed higher vascular invasion and marked pleomorphism. **(A)** Scanning magnification of FCBT hematoxylin and eosin (H&E) staining. **(B)** Scanning magnification of FCBT Masson staining (vascular invasion, black arrow). **(C)** Higher magnification of FCBT H&E staining. **(D)** Higher magnification of FCBT Masson staining. **(E)** Scanning magnification of CCBT H&E staining. **(F)** Scanning magnification of CCBT Masson staining. **(G)** Higher magnification of CCBT H&E staining. **(H)** Higher magnification of CCBT Masson staining.

The original histologic diagnosis was confirmed on archival hematoxylin and eosin (H&E) stained slides. All CBTs were classified as FCBT or CCBT according to H&E and Masson trichrome-stained slides, reviewed by two pathologists blinded to each other’s diagnosis. In case of disagreement, a third pathologist would be invited to make the final diagnosis (**Figure 1**). FCBT was defined as CBT with the presence of irregular cords and bands of hyalinized fibrous tissue that compartmentalized the lesion into irregular nests, islands, or cords of tumor cells, imparting an infiltrative appearance (8). Tumor features, such as volume, vascular invasion, and marked pleomorphism, were noted.

### Outcomes

Primary outcomes (major adverse event [MAE]) included recurrence, occurrence of metastasis, stroke, and all-cause mortality; secondary outcomes were nerve injuries. Other complications considered were infection and nerve injuries related to glossopharyngeal (IX), vagus (X)/recurrent laryngeal, superior laryngeal, accessory (XI) nerves, hypoglossal (XII) nerves, and sympathetic ganglion. The complications that had not resolved at postoperative discharge were included in the analysis. Recurrence was defined as a tumor of the same pathologic type in the ipsilateral neck after complete (R0) resection. Metastatic tumors were defined as CBT with nodal involvement or systemic disease. The duration of follow-up was calculated from the date of the CBT resection to the date of cancer progression, last follow-up, or death.

### CBT Resection

Tumor extirpation was achieved by adequate exposure, along with proximal and distal vascular control. Associated cranial nerves (CNs) IX, X, XI, XII and the superior laryngeal nerve were identified and separated, whenever possible. Subsequently, arterial dissection along the periadventitial white line of Gordon from the “outside in” was performed, leaving the central area of the carotid bifurcation until last. In case of CCA/ICA wall resection, repair using a patch or interposition vein graft was performed (10).

### Statistical Analyses

Results were analyzed using SPSS V.23 (IBM Corp, Armonk, NY). Kaplan-Meier analyses for MAE, recurrence, all-cause mortality, and metastatic tumors were based on clinical imaging follow-up. For analysis, patients with tumor recurrence, systemic metastasis, or stroke at diagnosis were excluded, whereas patients who died or presented with recurrence, systemic metastasis, or stroke for the first time during the study period were considered. The statistical differences between Kaplan-Meier curves were determined using the log-rank test. Correlation statistics were calculated using logistic regression for binary dependent variables. The models were compared using logistic regression and McFadden R2 values, with values closer to 1 representing the superior model. Data were assessed for normality and expressed as number (%) for categorical variables and mean ± standard deviation for continuous variables. A two-tailed student t-test was used to analyze continuous variables. Categorical variables were compared using the chi-square test or Fisher’s exact test. A p-value of < 0.05 was considered statistically significant.

## Results

### Patient Demographics and Comorbidities

We studied a total of 236 unilateral CBT samples; 53 were FCBT and 183 were CCBT. No significant differences were observed between FCBT and CCBT patients in age, sex, smoking, or comorbidities including hypertension, diabetes, hyperlipidemia, and cardiovascular disease (**Table 1**). Forty-one patients (22.40%) in the CCBT group and eight patients (15.09%) in the FCBT group presented with symptoms such as tinnitus, pain, fainting, and dysphagia; there was no significant difference between the two groups.

**Table 1:** Demographics and procedural details of carotid body tumor patients.

	CCBT (n = 183) No. (%)	FCBT (n = 53) No. (%)			p-value	
Age, years	42.73 ± 12.36	42.07 ± 13.28			0.74	
Male	69 (37.70%)	22 (41.51%)			0.62	
Shamblin grade						
I	37 (20.22%)	9 (16.98%)			0.60	
II	112 (61.20%)	29 (54.72%)			0.40	
III	34 (18.58%)	15 (28.30%)			0.16	
Pre-operative embolization	1 (0.55%)	1 (1.89%)			0.35	
I	0	0			1.00	
II	0	1 (3.45%)			0.07	
III	1 (2.94%)	0			0.30	
Pre-operative stenting of the ICA	0	0			1.00	
I	0	0			1.00	
II	0	0			1.00	
III	0	0			1.00	
External carotid artery ligation	107 (58.47%)	36 (67.92%)			0.21	
Shamblin class I	0	1 (11.11%)			0.45	
Shamblin class II	85 (75.89%)	21 (72.41%)			0.67	
Shamblin class III	22 (64.71%)	14 (93.33%)			0.02	
Internal carotid artery reconstruction	35 (19.13%)	20 (37.74%)			0.013	
Shamblin class I	0	0			1.00	
Shamblin class II	14 (12.50%)	8 (27.59%)			0.04	
Shamblin class III	21 (61.76%)	12 (80.00%)			0.10	
Amount of EBL, cm^3^	311.69 ± 243.61	559.62 ± 491.55			0.00	
Shamblin class I	89.73 ± 73.10	273.33 ± 310.28			0.12	
Shamblin class II	323.84 ± 247.18	524.14 ± 465.72			0.00	
Shamblin class III	513.24 ± 404.95	800.00 ± 539.84			0.00	
Volume, cm^3^	17.03 ± 15.37	20.71 ± 13.77			0.12	
Shamblin class I	9.80 ± 7.69	10.27 ± 10.28			0.93	
Shamblin class II	15.00 ± 10.35	18.86 ± 10.88			0.17	
Shamblin class III	31.58 ± 24.24	30.55 ± 15.05			0.80	
Vascular invasion	13 (7.10%)	13 (24.53%)			0.007	
Shamblin class I	2 (5.40%)	1 (11.11%)			0.07	
Shamblin class II	7 (6.25%)	7 (24.14%)			0.005	
Shamblin class III	4 (11.76%)	5 (33.33%)			0.02	
Marked pleomorphism	8 (4.37%)	12 (22.64%)			0.003	
Shamblin class I	1 (2.70%)	2 (22.22%)			0.054	
Shamblin class II	5 (4.46%)	6 (20.69%)			0.00	
Shamblin class III	2 (5.88%)	4 (26.67%)			0.014	

Data are presented as n (%) or mean ± standard deviation (SD).

CCBT, conventional carotid body tumor; FCBT, fibrous carotid body tumor; EBL, established blood loss; ICA, internal carotid artery.

### Procedural Details

The procedural details of CBT resection are shown in **Table 1**. The distribution of Shamblin grades was similar between the two groups, where Shamblin class II CBTs accounted for more than half the cases. One patient with CCBT and one patient with FCBT received pre-operative embolization. No patient received pre-operative stenting of the ICA. ECA ligation was conducted in 107 patients with CCBT (58.47%) and 36 patients with FCBT (67.92%). The proportion of patients that underwent ECA ligation was similar between the FCBT and CCBT groups in Shamblin class I and II and was significantly higher for FCBTs in Shamblin class III (93.33% *vs.* 64.71%, p=0.02).

ICA reconstruction was required in 35 CCBT patients (19.13%) and 20 FCBT patients (37.74%); this difference was statistically significant (p=0.013). Specifically, no patient required ICA reconstruction in Shamblin class I, whereas a higher proportion required it in Shamblin class II or III FCBTs. However, this difference was significant only in Shamblin class II (27.59% *vs.* 12.50%, p=0.04).

Significantly higher established blood loss (EBL) was observed in the FCBT group than in the CCBT group (559.62 ± 491.55 *vs.* 311.69 ± 243.61, p<0.01), specifically in Shamblin class II and III CBT (Shamblin class II: 524.14 ± 465.72 *vs.* 323.84 ± 247.18, p<0.01; Shamblin class III: 800.00 ± 539.84 *vs.* 513.24 ± 404.95, p<0.01). The differences for Shamblin class I were insignificant.

### Tumor Characteristics

The volume of CBTs was similar between groups and increased with Shamblin grade. The volumes of Shamblin class I, II, and III tumors were 9.80 ± 7.69, 15.00 ± 10.35, and 31.58 ± 24.24 cm^3^ in the CCBT group, respectively, and 10.27 ± 10.28, 18.86 ± 10.88, and 30.55 ± 15.05 cm^3^ in the FCBT group, respectively.

Histological analysis revealed a significantly higher proportion of FCBTs than CCBTs exhibiting microscopic vascular invasion (24.53% *vs.* 7.1%, p<0.01) and marked pleomorphism (22.64% *vs.* 4.37%, p<0.01). The proportion was significantly higher in Shamblin class II (vascular invasion: 24.14% *vs.* 6.25%, p<0.01; marked pleomorphism: 20.69% *vs.* 4.46%, p<0.01) and III (vascular invasion: 33.33% *vs.* 11.76%, p=0.02; marked pleomorphism: 26.67% *vs.* 5.88%, p=0.01), but not in Shamblin class I (**Table 1**).

### Peri-Operative Outcomes

Stroke occurred in two patients postoperatively (Shamblin class II FCBT and Shamblin class III CCBT). The patient with Shamblin class III CCBT died of stroke after CBT resection. Overall, no significant difference was noted between the FCBT and CCBT groups in stroke incidence or mortality.

Total nerve injuries occurred in 26 of the 53 operations (49.06%) in the FCBT group and in 39 of the 183 operations (21.31%) in the CCBT group. The proportion of nerve injury was significantly higher in the FCBTs than in the CCBTs (p<0.01). Nerve injury rate increased with Shamblin grade in both groups. A significant difference between CCBT and FCBT was observed in Shamblin class II CBT (51.72% *vs.* 20.54%, p<0.01). For patients with nerve injury, neurotrophic drugs, supportive treatments, and symptomatic treatments were employed, and most of the nerve injury was well tolerated. Since this study only reviewed patients with unilateral CBT, seldom bilateral nerve injuries were observed, and no patients received invasive treatment such as tracheotomy.

The occurrence of CN X, XII, and superior laryngeal nerve injury was significantly higher in the FCBTs than in the CCBTs. No significant differences were noted between groups in accessory nerve (CN XI) injury, glossopharyngeal nerve (CN IX) injury, sympathetic ganglion injury, and infection rate. In Shamblin class I CBTs, no significant difference was observed in the aforementioned complications between the two groups. In Shamblin class II CBTs, the occurrence of CN X, XII, superior and recurrent laryngeal nerve injury, and infection was significantly higher in the FCBTs. In Shamblin class III CBTs, the occurrence of glossopharyngeal nerve (CN IX) injury and superior laryngeal nerve injury was significantly higher in the FCBT group (**Table 2**).

**Table 2:** Postoperative and follow-up outcomes after surgical excision of carotid body tumor.

Outcome	CCBT (n = 183) No. (%)	FCBT (n = 53) No. (%)	p-value
In-hospital death	1 (0.55%)	0	0.59
Shamblin class I	0	0	1.00
Shamblin class II	0	0	1.00
Shamblin class III	1 (2.94%)	0	0.15
In-hospital stroke	1 (0.55%)	1 (1.89%)	0.35
Shamblin class I	0	0	1.00
Shamblin class II	0	1 (3.45%)	0.07
Shamblin class III	1 (2.94%)	0	0.30
Total nerve injury	39 (21.31%)	26 (49.06%)	0.00
Shamblin class I	3 (8.11%)	2 (22.22%)	0.37
Shamblin class II	23 (20.54%)	15 (51.72%)	0.00
Shamblin class III	13 (38.24%)	9 (60.00%)	0.10
Glossopharyngeal nerve injury (CN IX)	1 (0.55%)	2 (3.77%)	0.24
Shamblin class I	0	0	1.00
Shamblin class II	1 (0.89%)	0	0.69
Shamblin class III	0	2 (13.33%)	0.00
Vagus (CN X)/recurrent laryngeal nerve injury	10 (5.46%)	13 (24.53%)	0.00
Shamblin class I	1 (2.70%)	2 (22.22%)	0.07
Shamblin class II	5 (4.46%)	7 (24.14%)	0.00
Shamblin class III	4 (11.76%)	4 (26.67%)	0.10
Accessory nerve injury (CN XI)	0	0	1.00
Shamblin class I	0	0	1.00
Shamblin class II	0	0	1.00
Shamblin class III	0	0	1.00
Hypoglossal nerve injury (CN XII)	16 (8.74%)	12 (22.64%)	0.03
Shamblin class I	1 (2.70%)	0	0.82
Shamblin class II	10 (8.93%)	7 (24.14%)	0.02
Shamblin class III	5 (14.71%)	3 (33.33%)	0.06
Superior laryngeal nerve injury	10 (5.46%)	12 (22.64%)	0.006
Shamblin class I	0	1 (11.11%)	0.29
Shamblin class II	7 (6.25%)	7 (24.14%)	0.00
Shamblin class III	3 (8.82%)	4 (26.67%)	0.04
Sympathetic ganglion injury	7 (3.83%)	3 (5.66%)	0.56
Shamblin class I	1 (2.70%)	0	0.72
Shamblin class II	3 (2.68%)	2 (6.90%)	0.32
Shamblin class III	3 (8.82%)	1 (6.67%)	0.73
Infection	0	1 (1.89%)	0.32
Shamblin class I	0	0	1.00
Shamblin class II	0	1 (3.45%)	0.07
Shamblin class III	0	0	1.00
Follow-up period	62.21 ± 40.36	63.70 ± 41.37	0.81
All-cause mortality	1 (0.55%)	1 (1.89%)	0.35
Shamblin class I	0	0	1.00
Shamblin class II	0	0	1.00
Shamblin class III	1 (2.94%)	1 (6.67%)	0.19
Recurrence	0	2 (3.77%)	0.16
Shamblin class I	0	0	1.00
Shamblin class II	0	1 (3.45%)	0.07
Shamblin class III	0	1 (6.67%)	0.02
Metastasis	0	1 (1.89%)	0.32
Shamblin class I	0	0	1.00
Shamblin class II	0	0	1.00
Shamblin class III	0	1 (6.67%)	0.02
Major adverse event	1 (0.55%)	4 (7.55%)	0.06
Shamblin class I	0	0	1.00
Shamblin class II	0	2 (6.90%)	0.02
Shamblin class III	1 (2.94%)	2 (13.33%)	0.02

Data are presented as n (%) or mean ± standard deviation (SD).

CCBT, conventional carotid body tumor; CN, cranial nerve; FCBT, fibrous carotid body tumor.

### Follow-Up Results

**Figure 2:**
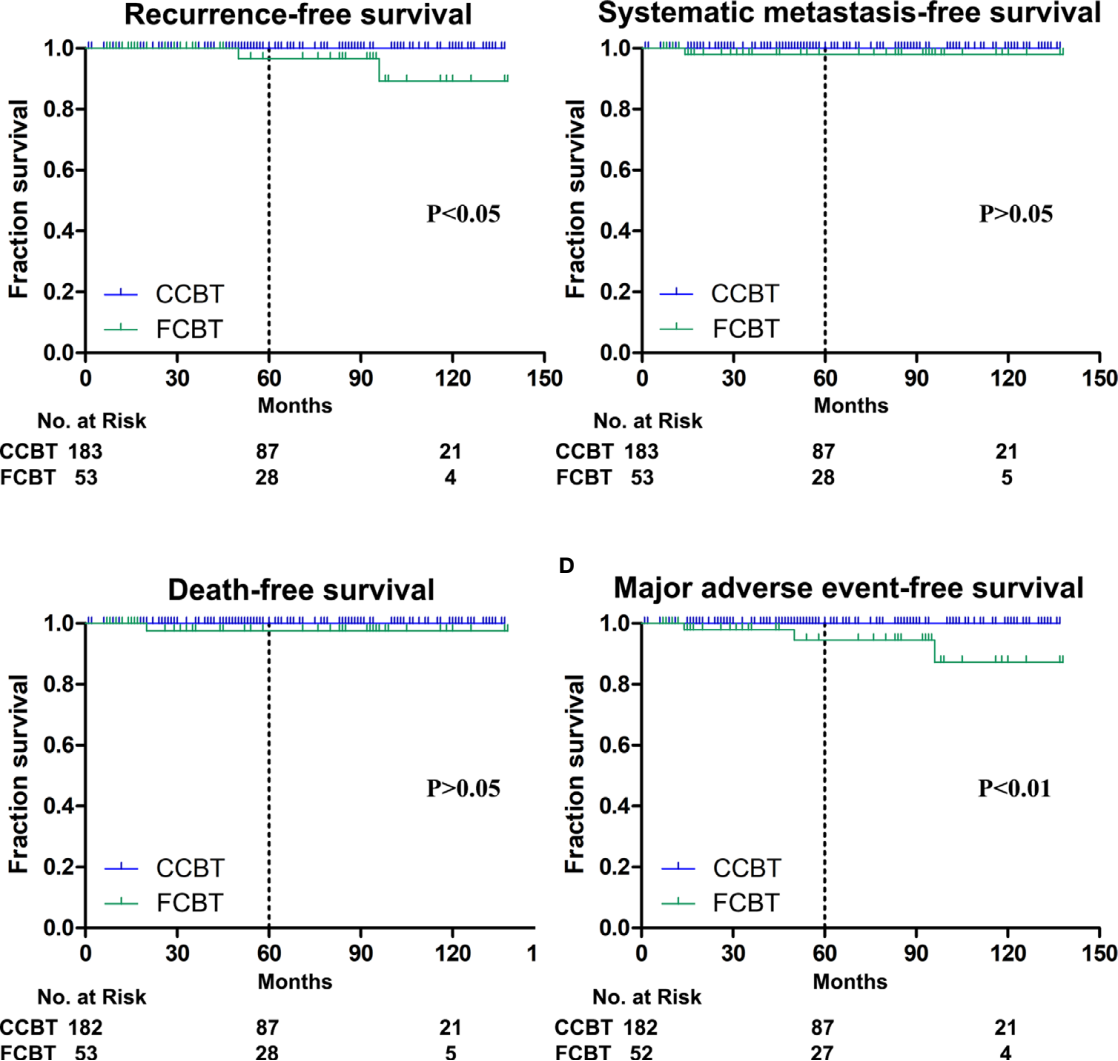
Kaplan-Meier plots. **(A)** Recurrence-free survival at 10 years was significantly lower in the fibrous carotid body tumors (FCBTs). **(B)** Systemic metastasis-free survival at 10 years was not significantly different between both groups. **(C)** Death-free survival at 10 years was not significantly different between both groups. **(D)** Major adverse event-free survival at 10 years was significantly lower in the FCBTs.

One FCBT of Shamblin Class II recurred 50 months after resection, and the other of class III recurred after 96 months. Therefore, a significantly higher proportion had tumor recurrence in Shamblin class III FCBTs compared with that in Shamblin class III CCBTs (**Table 2**). Both cases were re-operated for recurrent CBTs and remained free of further recurrence. The 1-, 5-, and 10-year recurrence-free survival was 100%, 96.7%, and 89.2%, respectively, in the FCBTs and 100%, 100%, and 100%, respectively, in the CCBTs. Recurrence-free survival at 10 years was significantly lower in the FCBTs (p<0.05, **Figure 2A**).

Iliac bone metastasis was observed 14 months after R0 resection in a patient with Shamblin class III FCBT, for which radiotherapy was performed. Therefore, a significantly higher metastatic rate was observed in Shamblin class III FCBTs, although the overall metastasis was not significantly different between the two groups (**Table 2**). The 1-, 5-, and 10-year systemic metastasis-free survival was 100%, 98.0%, and 98.0%, respectively, in the FCBTs and 100%, 100%, and 100%, respectively, in the CCBTs. Systemic metastasis-free survival at 10 years was not significantly different between the two groups (**Figure 2B**).

Two patients with Shamblin class III CBT died during follow-up. As mentioned above, one died of stroke, whereas the other of systemic metastasis after resection. The all-cause mortality rate was similar between the two groups in different Shamblin grades (**Table 2**). The 1-, 5-, and 10-year all-cause mortality-free survival was 100%, 97.6%, and 97.6%, respectively, in the FCBTs and 100%, 100%, and 100%, respectively, in the CCBTs. All-cause mortality-free survival at 10 years was similar between the two groups (**Figure 2C**).

MAE rate was higher in the FCBT group, but not statistically significant (7.55% *vs.* 0.55%, p=0.06). MAE occurred significantly more in FCBTs than in CCBTs of Shamblin class II (6.9% *vs.* 0, p=0.02) and III (13.33% *vs.* 2.94%, p=0.02) but not Shamblin class I (**Table 2**). The 1-, 5-, and 10-year MAE-free survival was 100%, 94.5%, and 87.3%, respectively, in the FCBTs and 100%, 100%, and 100%, respectively, in the CCBTs. MAE-free survival at 10 years was significantly lower in the FCBTs (p<0.01, **Figure 2D**).

### Comparison of Logistic Models

The Shamblin grade and pathological fibrosis were correlated with nerve injury, and pathological fibrosis was correlated with MAE (**Table 3**). The logistic model using Shamblin grade alone showed McFadden’s pseudo R^2^ statistics of 0.275, 0.061, 0.245, 0.061, 0.101, and 0.052 in predicting all-cause mortality, recurrence, metastasis, stroke, MAE, and nerve injury, respectively. The logistic model using pathological fibrosis alone showed McFadden’s pseudo R^2^ statistics of 0.032, 0.262, 0.232, 0.032, 0.158, and 0.053 in predicting all-cause mortality, recurrence, metastasis, stroke, MAE, and nerve injury, respectively. The logistic model using both pathological fibrosis and Shamblin grade showed McFadden’s pseudo R^2^ statistics of 0.290, 0.304, 0.431, 0.086, 0.237, and 0.099 in predicting all-cause mortality, recurrence, metastasis, stroke, MAE, and nerve injury, respectively. The increased McFadden’s pseudo R^2^ values demonstrated that the combination of pathological fibrosis and Shamblin grade is a better predictor of all-cause mortality, recurrence, metastasis, and MAE (**Table 4**).

**Table 3:** Correlation between tumor characteristics and outcome variables.

Outcome	Shamblin grade	Pathological fibrosis
	Regression (p)	Regression (p)
All-cause mortality	0.994	0.554
Recurrence	0.415	0.995
Metastasis	0.994	0.995
Stroke	0.334	0.451
Major adverse event	0.087	0.026
Total nerve injury	0.001	0.000

**Table 4:** Comparison of logistic models.

Outcome 1: All-cause mortality	McFadden R^2^
Model 1a: All-cause mortality = Shamblin	0.275
Model 1b: All-cause mortality = Pathological fibrosis	0.032
Model 1c: All-cause mortality = Shamblin, Pathological fibrosis	0.290
Outcome 2: Recurrence	
Model 2a: Recurrence = Shamblin	0.061
Model 2b: Recurrence = Pathological fibrosis	0.262
Model 2c: Recurrence = Shamblin, Pathological fibrosis	0.304
Outcome 3: Metastasis	
Model 3a: Metastasis = Shamblin	0.245
Model 3b: Metastasis = Pathological fibrosis	0.232
Model 3c: Metastasis = Shamblin, Pathological fibrosis	0.431
Outcome 4: Stroke	
Model 4: Stroke = Shamblin	0.061
Model 4: Stroke = Pathological fibrosis	0.032
Model 4: Stroke = Shamblin, Pathological fibrosis	0.086
Outcome 5: Major adverse event	
Model 5a: Major adverse event = Shamblin	0.101
Model 5b: Major adverse event = Pathological fibrosis	0.158
Model 5c: Major adverse event = Shamblin, Pathological fibrosis	0.237
Outcome 6: Total nerve injury	
Model 6: Total nerve injury = Shamblin	0.052
Model 6: Total nerve injury = Pathological fibrosis	0.053
Model 6: Total nerve injury = Shamblin, Pathological fibrosis	0.099

## Discussion

Extensive fibrosis is an unusual morphologic variant of CBTs (8). Here, we analyzed the association between Shamblin classification and fibrosis of CBT and the clinical outcomes in a cohort of 236 patients who underwent CBT resection. Compared with CCBT, FCBT tended to present with invasive histological characteristics such as increased vascular invasion and marked pleomorphism. During the operation, the occurrence of EBL and nerve injury, particularly CN X, XII, superior and recurrent laryngeal nerve injury, was significantly higher in patients with FCBT. Pathological fibrosis resulted in significantly decreased 10-year postoperative recurrence-free and MAE-free survival. Furthermore, the addition of pathological fibrosis supplemented the Shamblin grade for improved prediction of all-cause mortality, recurrence, metastasis, and MAE compared to the Shamblin grade alone.

An important finding was the relatively poor clinical outcomes in patients with FCBT compared to those with CCBT. CBTs with prominent fibrosis were first described in 2006 by Plaza et al. (8); however, the impact of pathological fibrosis on postoperative complications or outcomes has not been studied (8, 9). Carotid body paragangliomas are predominantly benign tumors with a combined ipsilateral and contralateral recurrence rate of 4.2% and a malignancy rate of 5% (11, 12). Here, the overall ipsilateral recurrence, metastasis, and all-cause mortality rates were 0.85%, 0.42%, and 0.85%, respectively. The recurrence and metastasis rates were lower in our study as we routinely excised the surrounding abnormal lymph nodes as advocated by Kruger et al. (13). All tumor recurrence and metastasis occurred in the FCBT group, and pathological fibrosis combined with the Shamblin grade improved the prediction of recurrence, metastasis, and MAE risk. However, results were insignificant, which could be owing to the low MAE rate, including stroke, mortality, recurrence, and metastasis. The 10-year MAE-free and ipsilateral tumor recurrence-free survival rates in the FCBTs were 87.3% and 89.2%, respectively; these were significantly higher than those in the CCBTs. We conjecture that our results may be due to the increased vascular invasion and marked pleomorphism in FCBT, which complicated FCBT resection and strokes caused by inadvertent ICA manipulation (14).

One of the most fatal postoperative complications is stroke. In a meta-analysis of CBT excision in 4,061 patients (92 series), a clear association was observed between Shamblin status and rates of peri-operative stroke (5). Here, we observed a similar association between Shamblin status and rates of peri-operative stroke (Shamblin I, 0%; Shamblin II, 0.71%; Shamblin III, 2.04%), although the results were statistically insignificant. The incidence of stroke was significantly increased by ICA manipulation, including reconstruction, ligation, and repair of injury (14, 15). The Shamblin classification is widely used by surgeons to predict the need for arterial reconstruction (3); however, additional tumor characteristics are considered (16). In this study, significantly more patients required ICA reconstruction in the FCBTs than in the CCBTs, and more strokes occurred in the FCBTs than in the CCBTs although with no statistical significance (1.89% *vs.* 0.55%, p=0.35), which implied that pathological fibrosis might contribute to postoperative stroke after CBT resection. Sanna M et al. found that stenting of the ICA facilitates complete tumor removal with arterial preservation (17). Therefore, pre-operative stenting of the ICA may be useful to avoid strokes in patients with Shamblin III or fibrous CBT.

Inadvertent ICA manipulation caused by bleeding is strongly associated with postoperative stroke (14). We observed significantly more blood loss in the FCBT group. Patients with tumors with higher Shamblin classifications have been reported to experience greater blood loss and increased need for vascular sacrifice and reconstruction (18–20). Indeed, we observed increased blood loss and ICA reconstruction for CBTs with higher Shamblin classification, but more importantly, we recorded significantly greater blood loss and ICA reconstruction in the FCBTs than in the CCBTs of Shamblin class II and III. Prudent preoperative planning, particularly in patients with prominent pathological fibrosis or Shamblin class II and III CBTs, may avoid inadvertent ICA manipulation and bleeding. Pre-operative ECA embolization or ICA/CCA coverage with stent grafts have been proposed to reduce bleeding (17, 21). We preferred early ECA division during the operation in patients with prominent fibrosis or class II and III tumors, which was more effective than preoperative percutaneous embolization for reducing stroke rate (14).

CNIs are generally not considered serious complications, but they can be extremely disabling to the patient and should not be underestimated (5). CN deficits have been reported to occur in 11–49% of patients after CBT resection (22–26). The Shamblin classification is predictive of the risk of CNI (27). Consistent with previous studies, patients with higher Shamblin grade tumors presented with a higher incidence of CNI in our study (5, 18–20). Here, the occurrence of CNI was higher in the FCBTs than in the CCBTs, especially in Shamblin II CBTs. It is not uncommon to observe these CNs (and occasionally the glossopharyngeal nerve) to be either adherent to the CBT or encased within part of the CBT (5). The increased CNI rate may be due to the potential invasiveness and non-respectability caused by compression or erosion of local structures in FCBTs (8). Consistent with previous reports, the most frequently injured nerve after CBT resection was the hypoglossal nerve, followed by the vagus/recurrent laryngeal nerve and the superior laryngeal nerve (5). Pathological fibrosis significantly increased the injury rates of these three nerves. Hypoglossal nerve injury results in tongue deviation, vagus/recurrent laryngeal nerve injury results in hoarseness, and superior laryngeal nerve injury results in dyspepsia. While the prediction of complications is important for decision making, preoperative planning, and for obtaining the informed consent, prevention of complications is even more important. Meticulous surgical techniques and timely operations in the initial stages of FCBTs are the optimal way to minimize complications (27).

It has been suggested that all CBTs should be viewed as having malignant potential (12). Some authors hold the opinion that the presence of prominent stromal fibrosis and hyalinization, results in a pseudo-infiltrative histologic growth pattern, which may be mistaken for an aggressive malignant neoplasm (8). Our study demonstrated that vascular invasion and marked pleomorphism were significantly greater in FCBT than in CCBT, highlighting the resemblance to invasive malignancy (28). Recurrent tumors significantly increase the accompanying risk to surrounding nerves during dissection (13). Therefore, we propose that radical resection and sampling of the tumor and regional lymph nodes, regardless of their size or clinical symptoms, result in safer operations; earlier intervention is justified to reduce the risk of disease recurrence and metastasis in patients with FCBT. Moreover, timely surgery combined with radiotherapy may be beneficial for FCBTs with vascular invasion or marked pleomorphism (29). Nevertheless, the benefits of radiotherapy combined with surgery in the treatment of paragangliomas remains controversial (29). Further, closer clinical surveillance of patients with FCBT may be beneficial for recurrent or metastatic tumor detection.

A limitation of this study is its retrospective nature. Additionally, it is recommended that patients be tested for the *SDHx* mutation, as this mutation is thought to reflect a subgroup of patients with CBTs requiring additional tests and preoperative planning (12). As only five patients received tests for this mutation, we did not consider the impact of this mutation on postoperative outcomes or complications. Furthermore, only unilateral CBT patients with paraffin-embedded tissue available were included in this study, which may have resulted in selection bias. Finally, we were only able to obtain precise fibrosis status of patients based on postoperative histological analysis, as preoperative examination was not feasible. However, the development of magnetic resonance imaging (MRI) and magnetic resonance elastography may be useful for the quantitative evaluation of tumor stiffness and perfusion in humans (30), and collagen-targeted molecular MRI can detect tumor fibrosis in mice (31), which makes identifying pathological fibrosis in patients with CBT possible. In this study, we associated one of the CBT pathological features, fibrosis, with prognosis after resection. Further studies are recommended for CBT pathological features, prognosis after resection, and MRI-based radiomics analysis.

In conclusion, pathological fibrosis in patients with CBT is important because of the poorer clinical outcomes and higher nerve injury in FCBT than in CCBT patients. Early surgical resection, routine excision of surrounding abnormal lymph nodes, and closer clinical surveillance in patients with FCBT are recommended. Development of effective approaches for preoperative identification of pathological fibrosis in patients with CBT is warranted.

## Data Availability Statement

The original contributions presented in the study are included in the article/supplementary material. Further inquiries can be directed to the corresponding authors.

## Ethics Statement

The studies involving human participants were reviewed and approved by Institutional Review Board of Zhongshan hospital, Fudan University. The patients/participants provided their written informed consent to participate in this study.

## Author Contributions

Conception and Design: HT, XT, XJ, and DG. Analysis and Interpretation: HT, SX, WF, and DG. Data Collection: HT, SX, XT, and XJ. Manuscript Writing: HT, XT, XJ, and DG. Critical Revision: HT, XT, XJ, WF, and DG. Final approval of the article: HT, XT, XJ, SX, WF, and DG. Statistical analysis: HT, XT, XJ, and DG. Funding acquisition: HT and DG. Overall responsibility: HT, XT, XJ, and DG. All authors contributed to the article and approved the submitted version.

## Funding

This work was sponsored by grants from the Shanghai Sailing Program (Grant No. 20YF1406700), grants from the National Natural Science Foundation of China (Grant No. 81970408) and Science and Technology Commission of Shanghai Municipality (Grant No. 19411966900).

## Conflict of Interest

The authors declare that the research was conducted in the absence of any commercial or financial relationships that could be construed as a potential conflict of interest.

## Abbreviations

CBT, Carotid body tumor; CCA, common carotid artery; CCBT, conventional carotid body tumors; CN, cranial nerve; EBL, established blood loss; ECA, external carotid artery; FCBT, fibrous carotid body tumor; H&E, hematoxylin and eosin; ICA, internal carotid artery; MRI, magnetic resonance imaging; SDHx, subunits of succinate dehydrogenase.
